# Pena–Shokeir syndrome's first case report from Syria

**DOI:** 10.1002/ccr3.6662

**Published:** 2022-11-27

**Authors:** Mohammad Badr Almoshantaf, Hidar Alibrahim, Haidara Bohsas, Shorouk Alabdo, Abdulqadir J. Nashwan, Sarya Swed

**Affiliations:** ^1^ Department of Neurosurgery Ibn Al‐Nafess Hospital Damascus Syria; ^2^ Faculty of Medicine Aleppo University Aleppo Syria; ^3^ Department of Pediatrics University Aleppo Hospital Aleppo Syria; ^4^ Nursing Department Hamad Medical Corporation Doha Qatar

**Keywords:** atrial septal defect (ASD), congenital anomalies, Pena–Shokeir syndrome (PSS)

## Abstract

Pena–Shokeir syndrome is considered to be a fatal congenital condition that is rarely diagnosed in neonates. We present the first‐ever reported case of Pena–Shokeir syndrome from Syria. Clinical assessment and early prenatal diagnosis are both needed to give the mother and baby more realistic options.

## Graphical abstract

There is no current treatment for babies with Pena–Shokeir syndrome. Key management includes palliative and supportive treatment. Although PSS is rare, doctors should always be aware of the possibility of a new incident.

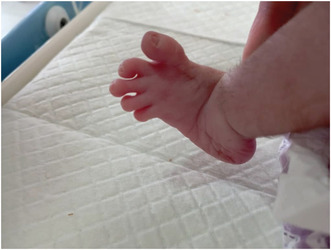

## 1 INTRODUCTION

Pena–Shokeir syndrome (PSS) is a deadly condition of multiple congenital contractures.^1^ It was first identified in 1974, and the estimated frequency is 1 in 12,000 births.^2^ PSS has been classified into 2 types: Type 1 is a fetal akinesia/hypokinesia sequence that is characterized by multiple joint contractures, facial anomalies, and pulmonary hypoplasia. Type 2—also known as cerebro‐oculo‐facio‐skeletal (COFS) syndrome—is a rapidly progressive neurological disorder resulting in brain atrophy, characterized by intracerebral calcifications, cataracts, microcornea, optic atrophy, progressive joint contractures, and growth failure.^3^ This syndrome may be difficult to prenatally diagnosed since it has comparable ultrasonographic characteristics with other disorders.^4^ However, as early as 12 weeks of gestation, ultrasonography may detect PSS by analyzing patterns, such as hypoplasia of the lung tissue, reduced intrauterine movement, fetal edema, and locked limb posture. Extremities might have been stretched or contracted; the knees are often extended, the elbows are flexed, and the feet may have a substantial rocker‐bottom or equinovarus deformity. Hypertelorism, a sunken nose tip, micrognathia, and low‐set ears are among the facial traits.^3^, ^5^ Although the eventual prognosis of PSS depends on the etiology, this syndrome has been nearly consistently fatal. Thirty percent of the fetuses affected by PSS are stillborn, and live‐born infants often die after about a month of their lives. A congenital cerebral anomaly or severe respiratory failure related to pulmonary hypoplasia is the most common cause of mortality in infants born prematurely.^3^, ^6^, ^7^ Women who have previously given birth to a child affected by PSS should have strict fetal monitoring during future pregnancies. This will enable the early detection of any defects that may arise, despite the fact that the recurrence risk may range from 0% to 25%.^2^ We report the first case of PSS from Syria. We recommend that clinical physicians be aware of any abnormalities in ultrasonography during pregnancy in order to early recognize signs of PSS.

## 2 CASE PRESENTATION

**FIGURE 1:**
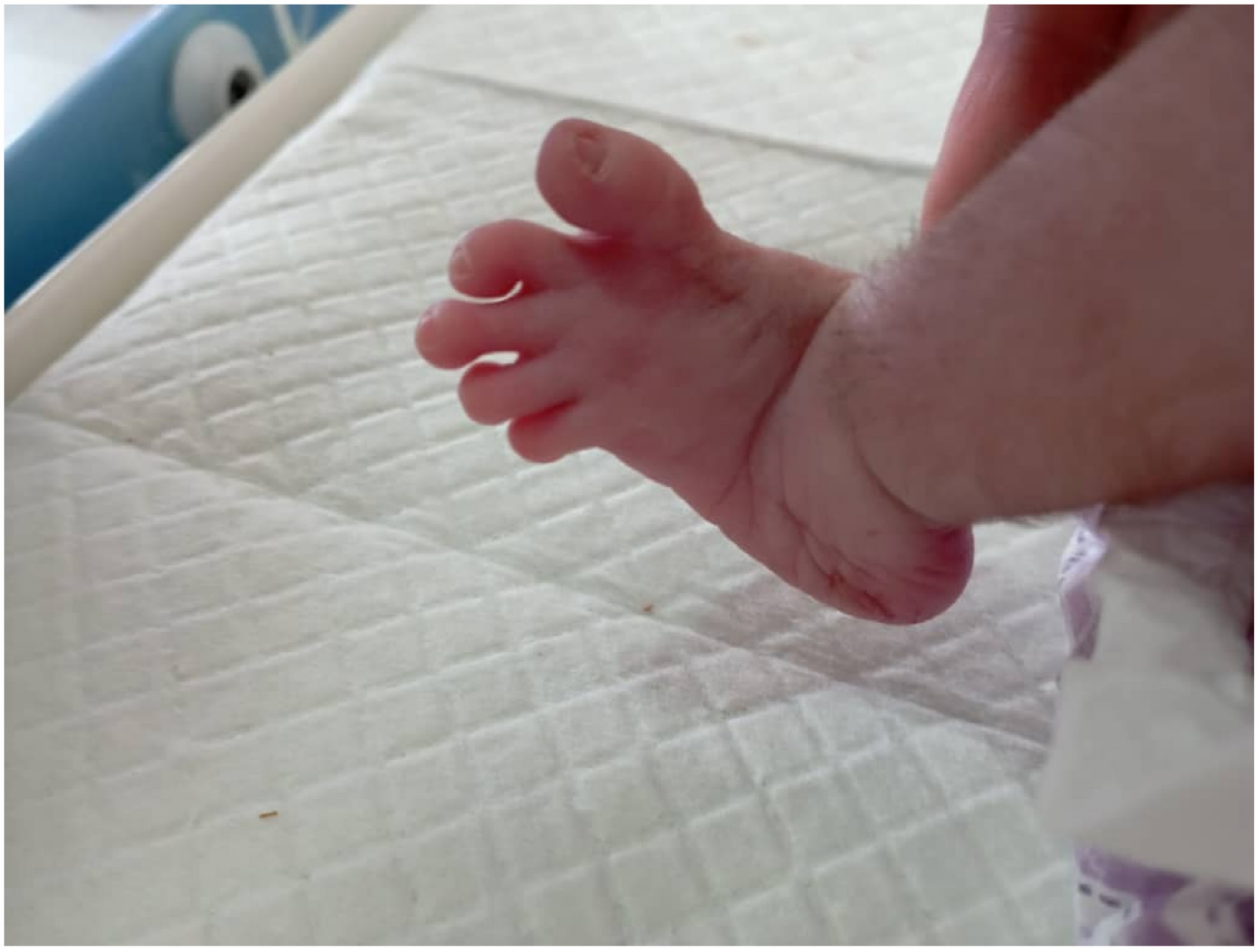
Rocker Bottom Foot

**FIGURE 2:**
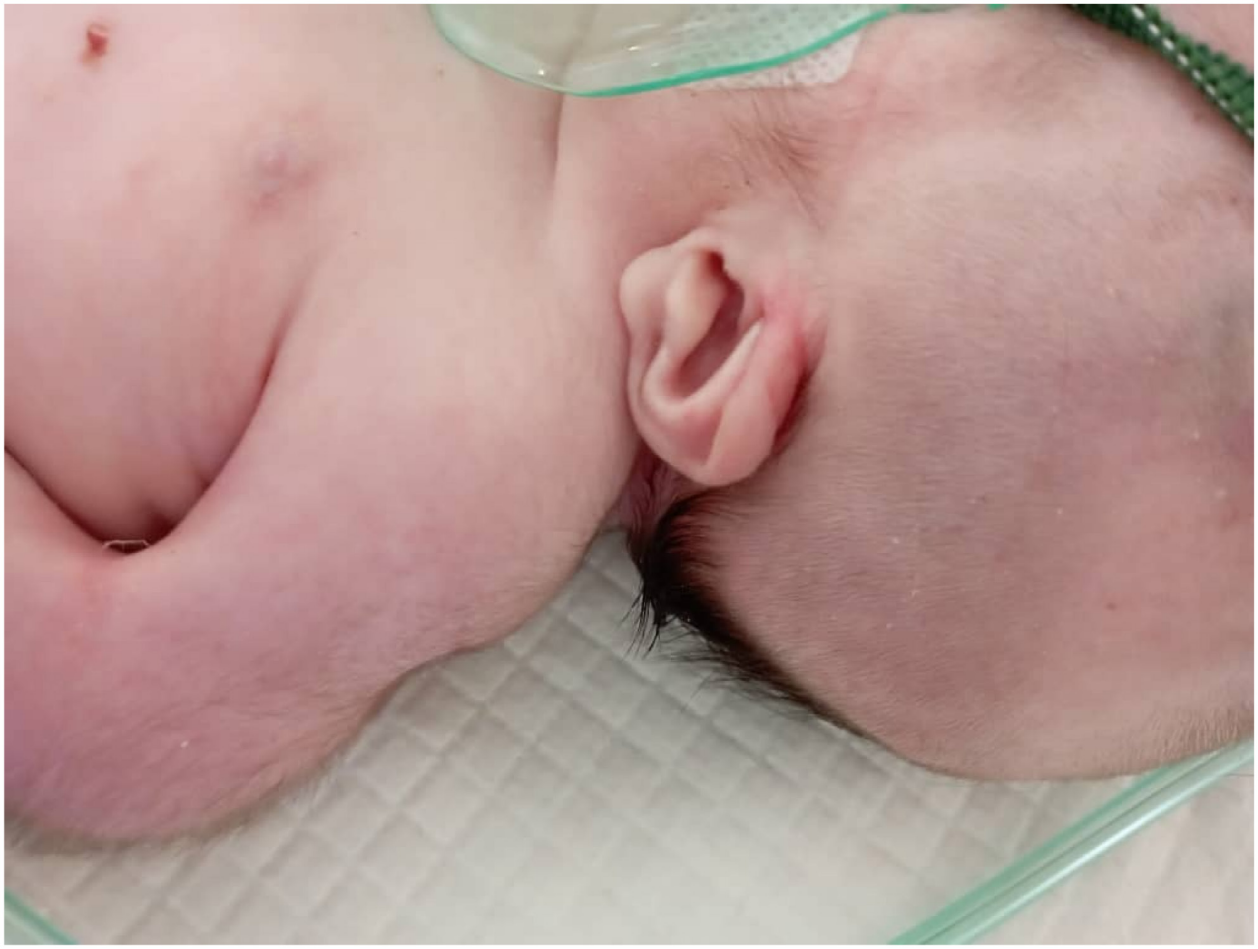
Large Ear

**FIGURE 3:**
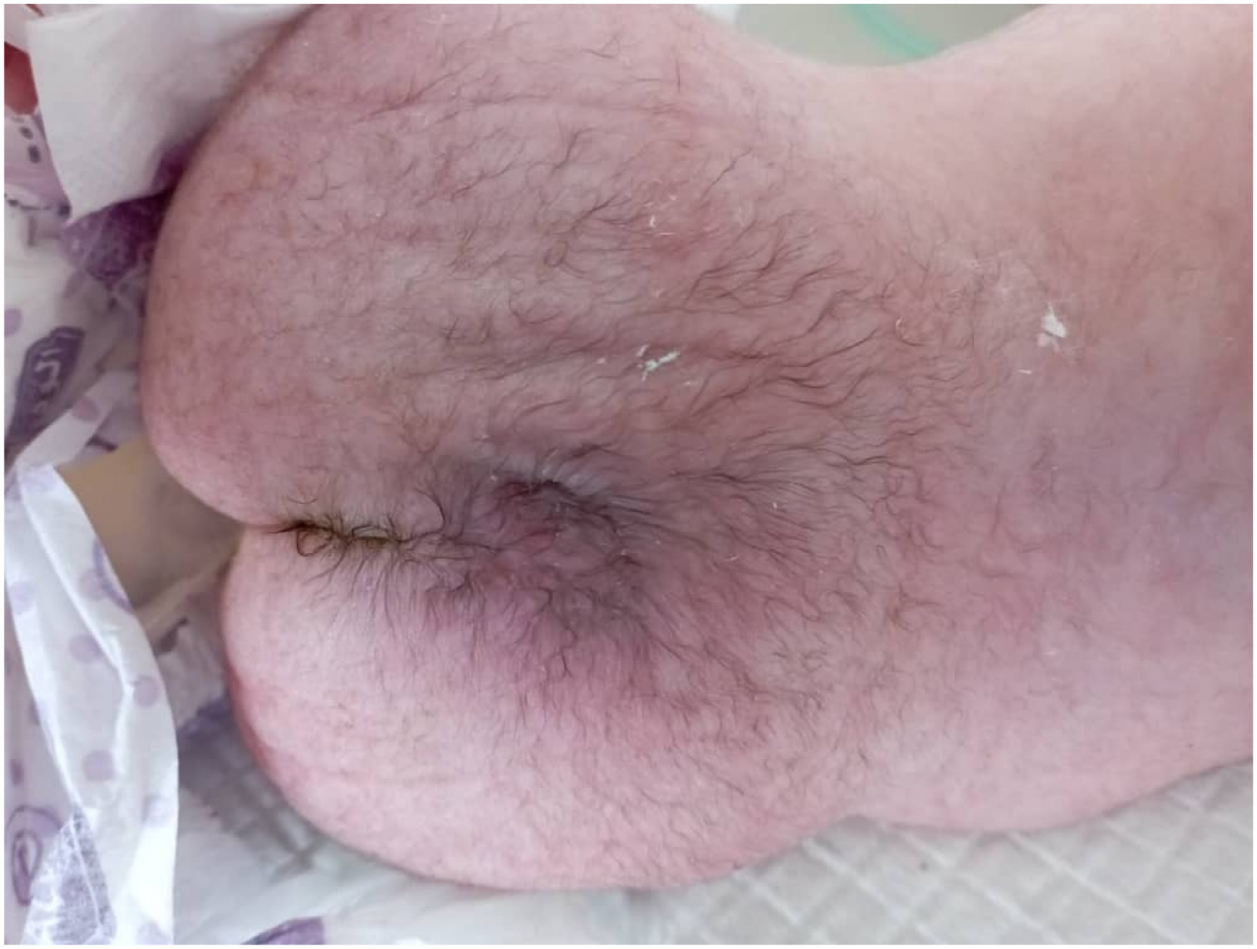
Hairy Back

We report a case of a female neonate who was diagnosed with PSS upon birth. The mother is a 33‐year‐old non‐consanguineous married woman. In her last pregnancy, she had a history of blood transfusions in the first trimester, hydramnios, and was absent of fetal movement. She underwent a cesarean delivery due to fetal‐pelvic maladjustment at the 37th gestational week. At birth, the female neonate was cyanosed and suffered from delayed screaming. Thus, the resuscitation protocol was initiated and successfully delivered. On the second day of birth, the neonate suffered from severe dyspnea with a Spo_2_ of 80%, a heart rate of 180, and a respiratory rate of 76. On clinical examination, the baby had multiple skeletal malformations like fixed flexion of hips and knees (rocker bottom foot (Figure 1)), very short neck, microcephaly (head perimeter of 32 cm), large ears (Figure 2), upper teeth, microstomia, cleft palate, hypotonia, absent reflexes, ambiguous genitalia, and a hairy back (Figure 3). No cardiac murmurs were reported, but cardiac echography found an atrial septal defect (ASD), tricuspid valve failure, and high pulmonary tension. The chest X‐ray was unremarkable. Depending on the clinical and morphological features observed, we diagnosed the neonate with PSS. Due to the overall bad respiratory condition, the neonate was admitted to the incubator unit with an oxygen mask. No signs of recovery were noted, and eventually, the baby died on the 3rd day of delivery due to cardiopulmonary failure as resuscitation was ineffective.

## 3 DISCUSSION

Pena–Shokeir syndrome (PSS) is a form of fetal development akinesia deformity that is characterized by early‐onset neurogenic arthrogryposis and hypoplastic lungs.^8^ Several studies have suggested that this syndrome is caused by mutations in the *RAPSN* and *DOK7* genes.^9^ In addition, autosomal dominant neuromuscular conditions can present with PSS when the gene defect is in the homozygous state.^3^ Many risk factors contribute to this disorder, including positive family history, environmental conditions, such as trauma, and hypotension.^10^ Despite various possible diagnoses, PSS is related to chromosome 18 trisomy, such as arthrogryposis and micrognathia.^2^ It is possible to diagnose PSS in the case of a normal chromosomal study, micrognathia, and arthrogryposis in children.^10^ Current molecular genetic studies have increased our understanding of the hereditary reasons for this syndrome, indicating that many patients are at the end of other identified neuromuscular diseases.^3^ Despite pulmonary hypoplasia being crucial for conclusively defining PSS, this finding is often seen in the latter stage of fetal akinesia.^11^ Magnetic resonance imaging (MRI) is another imaging technique for determining the presence of PSS. Fetal MRI should be requested even if it is not necessary to diagnose PSS when there is a suspicion of prenatal central nervous system defects. By comparing our case to other reported examples in the literature, we found a case reported by Adam et al.^5^ for a young pregnant woman who had a standard ultrasound scan at 24 weeks of pregnancy. The routine second‐trimester ultrasonography showed fetal micrognathia, a missing septum pellucidum, significant hyper‐lordosis, and decreased fetal movements. PSS was diagnosed based on prenatal ultrasound, MRI results, and normal fetal karyotype. The presence of pulmonary hypoplasia was revealed in postnatal ultrasonography with a lower ratio of the fetal lung to head circumference (LHR) = 0.62 corroborated the final diagnosis of PSS. According to Eduardo Santana et al.,^2^ another young nulliparous woman in her second pregnancy was hospitalized in the 28th week due to possible fetal arthrogryposis. The 2D ultrasonography revealed chronic spine hyperextension with head extension, persistent arm and leg flexion, hands and feet twisting, evidence of pulmonary hypoplasia, and retrognathia. The fetal position included continuous bending of the upper and lower limbs, hands and feet twisting, and more details of the micrognathia. During the fetal echocardiography test, a peri‐membranous interventricular septal defect with intermittent fetal bradycardia was seen.

Due to the poor prognosis of PSS during late pregnancy, termination may be offered as an option. Alternatively, the parents could choose complete resuscitative post‐delivery or supportive treatment after birth. Unfortunately, not all patients will have a prenatal diagnosis, and making decisions about lifesaving postpartum measures may be challenging. This article showed a rare case that causes severe abnormalities and requires constant follow‐up and monitoring to achieve the best management.

## 4 CONCLUSION

In this case, PSS has never before been reported in Syria. A professional understanding of this disease and early prenatal diagnosis is needed for better treatment choices for the mother and child. Since there is no cure for PSS, the main ways to help a baby with it are to keep an eye on them and give them palliative care.

## AUTHOR CONTRIBUTIONS

**Mohammad Badr Almoshantaf:** Writing – original draft; writing – review and editing. **Hidar Alibrahim:** Writing – original draft; writing – review and editing. **Haidara Bohsas:** Writing – original draft; writing – review and editing. **Shorouk Alabdo:** Writing – original draft; writing – review and editing. **Abdulqadir Nashwan:** Writing – original draft; writing – review and editing. **Sarya Swed:** Writing – original draft; writing – review and editing.

## CONFLICT OF INTEREST

All authors declared no conflict of interest.

## EHTICAL APPROVAL

This case report was reviewed and approved by the ethics committee, Aleppo University Hospital, Aleppo University, Syria.

## CONSENT

A written informed consent was obtained from the patient to publish this report in accordance with the journal's patient consent policy.

## GUARANTOR

D. Mohammad B. Almoshantaf.

## US SANCTIONS

I would like to declare that the authors have prepared this submission in their personal capacity and not as an official representative or otherwise on behalf of a sanctioned government.

## Data Availability

The data that support the findings of this study are available from the corresponding author upon reasonable request.
